# Exploring COVID-19 Case Fatality in Relation to the Prevalence of Chronic Conditions and Health Behaviors in Appalachian Kentucky

**DOI:** 10.13023/jah.0302.05

**Published:** 2021-05-03

**Authors:** W. Jay Christian

**Affiliations:** Department of Epidemiology, University of Kentucky

**Keywords:** Appalachia, COVID-19, BRFSS, geography, Kentucky

## Abstract

**Background:**

Research has demonstrated that common chronic conditions, especially those related to cardiovascular health, are important risk factors for severe COVID-19 symptoms or hospitalization. Population prevalence rates of such conditions have not previously been examined in relation to COVID-19 case fatality rates in the Central Appalachian region.

**Purpose:**

This study examined prevalence rates of selected chronic conditions and COVID-19 case fatality rates to determine whether the relationship between them is consistent across Appalachian and non-Appalachian regions of Kentucky.

**Methods:**

Data from Kentucky’s Behavioral Risk Factor Survey (KyBRFS) were used to calculate prevalence rates of asthma, diabetes, influenza vaccination, hypertension, obesity, having a personal doctor, physical inactivity, and cigarette smoking. Publicly available COVID-19 case and death counts by county were used to calculate incidence and case fatality rates. Units of analysis were 41 single- and multi-county areas developed to visualize KyBRFS prevalence rates. Analysis included t-tests to compare Appalachian and non-Appalachian regions, and correlations characterizing associations between COVID-19 case fatality and rates of chronic conditions and behaviors.

**Results:**

Incidence and case fatality rates for COVID-19 were slightly lower in the Appalachian region, but not significantly. Significant correlations between COVID-19 case fatality and the prevalence of chronic conditions and behaviors were more common in the non-Appalachian region.

**Implications:**

Case fatality rates in Appalachia appear lower than expected, given the high prevalence of important chronic conditions and behaviors known to be associated with poor COVID-19 outcomes. This phenomenon merits further research and should be considered by public health researchers when examining COVID-19 outcomes in Kentucky and neighboring states.

## BACKGROUND

Recent research has demonstrated that several common chronic conditions related to cardiovascular health—including diabetes, hypertension, and obesity—are important risk factors for severe COVID-19 symptoms or hospitalization due to COVID-19.^1^–^4^ Several of these conditions are also related to tobacco use, which varies widely in prevalence across the U.S. by geography, demography, and socioeconomic status.^5^,^6^ The Centers for Disease Control and Prevention (CDC) also asserts that individuals with moderate to severe asthma, which is associated with exposure to tobacco smoke, could be at greater risk for severe COVID-19.^7^

Researchers have yet to directly examine the relationship between COVID-19 case fatality rates and the prevalence of these chronic conditions and health-related behaviors in states with counties belonging to the Central Appalachian subregion, which includes 54 of Kentucky’s 120 counties, all of West Virginia, and smaller portions of Tennessee and Virginia. Although COVID-19 incidence has been generally lower in this region, examining case fatality is important because its population has very high prevalence rates of tobacco use and most of the chronic conditions mentioned above. Given these factors, it was hypothesized that COVID-19 case fatality rates would be higher in the Appalachian region and would be closely related to rates of chronic conditions and health-related behaviors associated with cardiovascular and respiratory health.

## METHODS

Geographic patterns of COVID-19 incidence and case fatality rates were visualized using choropleth maps, which display quantities among regions using shaded colors. Prevalence rates of select chronic conditions and behaviors from the Kentucky Behavioral Risk Factor Survey (KyBRFS)—Kentucky’s implementation of the Behavioral Risk Factor Surveillance System (BRFSS) survey—were examined in relation to COVID-19 case fatality rate using scatter plots, and Pearson’s correlation coefficients. These included asthma, diabetes, influenza vaccination, hypertension (i.e., high blood pressure), obesity (body mass index>30), having a personal doctor, physical inactivity (no physical activity in last 30 days), and cigarette smoking (current). The BRFSS is a system of telephone surveys conducted by all 50 states, the District of Columbia, and some United States territories. Coordinated by CDC, the BRFSS provides information on a great variety of health-related topics for over 400,000 Americans annually.^8^ The survey has a complex sampling design and responses are weighted to represent known proportions of age, race and ethnicity, sex, and geographic region.

Unfortunately, the limited number of responses to the KyBRFS survey produces highly unstable rates at the county level that are very likely to be inaccurate. In other words, there are not enough respondents in most of Kentucky’s 120 counties to produce reliable prevalence estimates. Therefore, prevalence rates for 16 counties with sufficient respondents to the survey, and 25 multi-county groups were used in this study. Counties with few respondents were aggregated to these multi-county groups based on similar socioeconomic and demographic characteristics (from the U.S. Census or American Community Survey) in a regionalization algorithm that required all counties in each group to be contiguous.^9^ These 41 county groups are used to report the prevalence of select BRFSS responses for small areas in Kentucky at the kentuckyhealthfacts.org website and comprised the units of analysis for this ecologic study.

**Figure 1:**
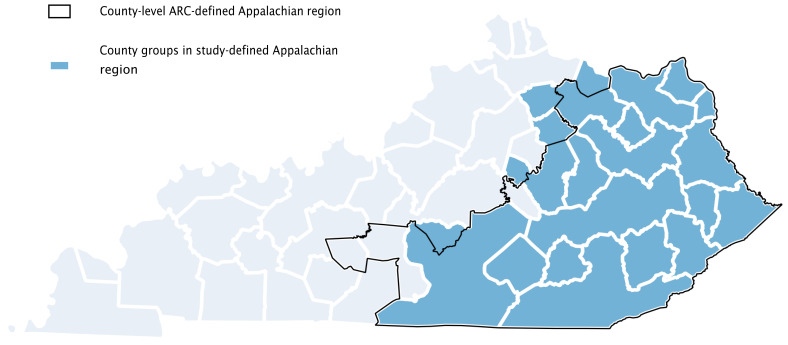
Definitions of Appalachian Kentucky: Appalachian Regional Commission (ARC) and This Study

Because Appalachian designation is conferred by the Appalachian Regional Commission (ARC) to individual counties,^10^ it was necessary to create an alternative county group-level Appalachian designation to examine potential disparities. A county group was included in this study’s definition of the Appalachian region if a majority of counties comprising that county group was designated as Appalachian by the ARC. Where the number of ARC-defined Appalachian and non-Appalachian counties in a county group was equal, the county group was included in Appalachia if the majority of its population lived in ARC-defined Appalachian counties. Figure 1 displays this discrepancy between the ARC Appalachian designation and that used for this study. There were 23 non-Appalachian county groups and 18 Appalachian county groups.

Combined survey data for 2017–2019 from the KyBRFS was used to calculate current prevalence rates of the selected chronic conditions and behaviors—asthma, diabetes, influenza vaccination, hypertension, obesity, having a personal doctor, physical inactivity, and cigarette smoking—for all 41 county groups, re-weighting responses and using survey weights according to CDC guidelines.^11^ Having a personal doctor was included to explore whether this basic indicator of healthcare accessibility from the KyBRFS might be associated with COVID-19 case fatality rates. Influenza vaccination was also included because research has shown it could be associated with lower COVID-19 mortality in elderly populations.^12^

The Johns Hopkins University Center for Systems Science and Engineering (CSSE) online repository at GitHub.com provided cumulative COVID-19 case and death counts at the county level on 1 March 2021.^13^ These counts were aggregated to county groups, along with 2019 population estimates from the U.S Census Bureau, and incidence was calculated per 100,000 residents and fatalities per 100 cases (i.e., %).

Open-source QGIS (version 3.10) software was used to create maps of COVID-19 incidence and case fatality, and Stata (version 15.1) software was used for statistical analysis and graphing. Statistical analysis initially consisted of independent sample t-tests to identify significant differences in prevalence of chronic conditions, and COVID-19 incidence and case fatality rates, between Appalachian and non-Appalachian regions of Kentucky. Independent sample t-tests are used to detect significant differences in the distributions of measurements or other values (here, COVID-19 incidence or case fatality rates) from two groups (here, Appalachian and non-Appalachian county groups).

Stratified scatter plots were created to visualize linear relationships between the BRFSS variables and COVID-19 case fatality among county groups, and Pearson correlation coefficients were calculated to quantify these relationships and determine statistical significance. Correlations and fitted lines were also produced for all county groups. Briefly, Pearson correlation coefficients, which range from −1.00 to 1.00, describe the amount of variation in one quantity that can be predicted by variation in another, related quantity. Further, a correlation coefficient describes the slope of a line that best fits through the set of points in a scatter plot, such that very high and low correlation coefficients have steeper slopes.

## RESULTS

Table 1 displays summary statistics for each of the chronic conditions or behaviors, as well as COVID-19 incidence and case fatality, for Appalachian and non-Appalachian regions. Table 1 also indicates the number of respondents in each county group, showing that the means and medians for the two groups are fairly similar, despite differences in maximums. This table demonstrates that the Appalachian region had significantly higher rates of asthma, diabetes, hypertension, obesity, having a personal doctor, and physical inactivity. Only influenza vaccination and smoking were not significantly different from the non-Appalachian region, although smoking was marginally higher (27.7% vs. 24.4%, p=0.08).

The incidence and case fatality rates for COVID-19 were quite similar among Appalachian and non-Appalachian regions (p=0.89 and p=0.58, respectively). Figures 2a and 2b display maps of COVID-19 incidence and case fatality by county group that enable visualization of these differences. Several single-county “county groups” with high incidence rates are discernible across the state in Figure 2a. There were more county groups in the Appalachian region with high incidence of COVID-19, but high and low rates were scattered throughout the state. Figure 2b shows that many county groups with the highest case fatality rates were in western Kentucky, including several along the southern border with Tennessee, while case fatality rates in the Appalachian region generally appeared similar to those in central and northern Kentucky.

**Figure 3:**
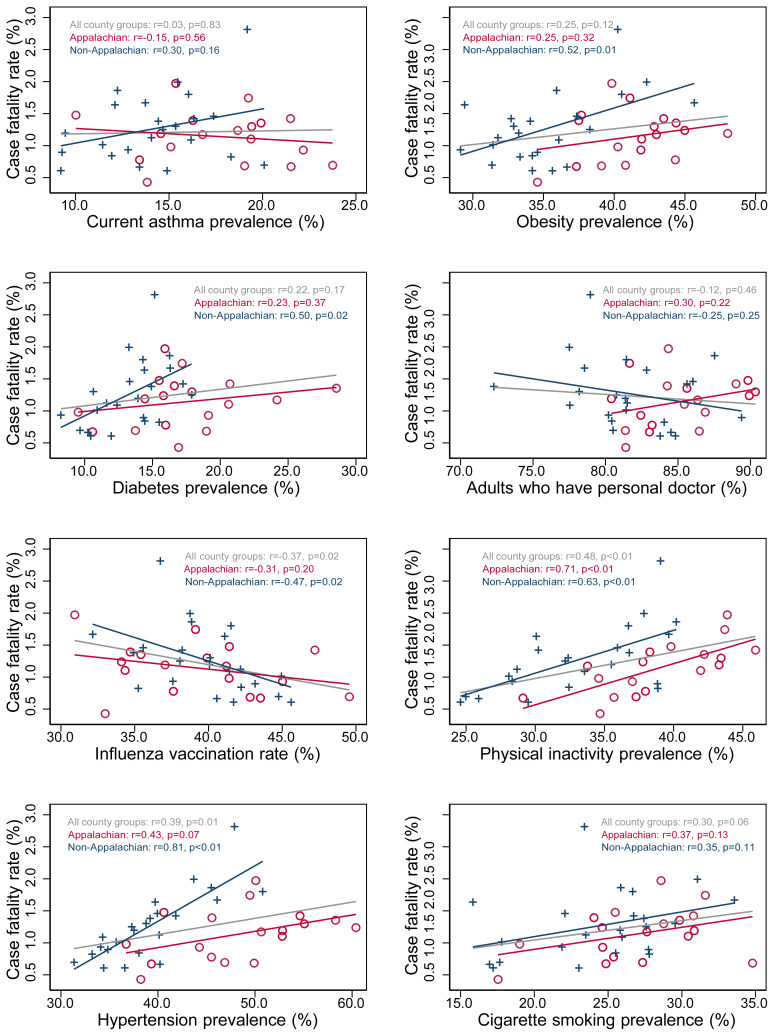
COVID-19 case fatality rates and prevalence of chronic conditions and behaviors by county group in Appalachian and non-Appalachian regions.

Scatter plots showing the relationships between COVID-19 case fatality and the selected conditions and behaviors appear in Figure 3 (COVID-19 case fatality rates and prevalence of chronic conditions and behaviors by county group in Appalachian and non-Appalachian regions. These plots (specifically, the gray fitted lines), and the accompanying correlation statistics, demonstrate that there were few significant relationships when all county groups were analyzed together. Note that most of the gray fitted lines are relatively flat—indicating very little correlation—compared to the blue and red lines. When non-Appalachian and Appalachian county groups were analyzed separately, relationships became quite apparent, especially among non-Appalachian county groups, which had steeper slopes for the fitted lines. In almost every graph, combined analysis produced smaller correlation coefficients and larger p-values when compared to non-Appalachian analysis. There were notable and statistically significant correlations between all chronic conditions related to the cardiovascular system and case fatality rates in the non-Appalachian region. Furthermore, prevalence rates of physical inactivity were significantly correlated with COVID-19 case fatality in the Appalachian region after stratification.

Regardless of stratification, there were no significant correlations observed between COVID-19 case fatality and prevalence rates of asthma, having a personal doctor, or cigarette smoking, although the last of these was nearly significant (r=0.30, p=0.06).

## IMPLICATIONS

Case fatality rates due to COVID-19 are not evenly distributed across the state. This study found that variation in cardiovascular health across regions of Kentucky could explain some of this difference. One of the most important findings of our analysis was that COVID-19 case fatality rates were not higher in the Appalachian region compared to the rest of the state. This was somewhat unexpected, given the high rates of conditions and behaviors known to increase risk for severe disease, and merits further investigation.

Analysis initially showed few significant relationships between prevalence rates of cardiovascular conditions and COVID-19 case fatality, but stratification by region demonstrated several substantial and statistically significant correlations in the non-Appalachian region. Furthermore, some correlations for the Appalachian region trended toward the expected direction, and the correlation with physical inactivity was stronger and significant. Still, there was little association between COVID-19 case fatality and the other chronic conditions in Appalachia when compared to the rest of the state.

It is unclear why some relationships that clearly manifested in the non-Appalachian region were not observed in Appalachia. It is possible that there are important determinants of COVID-19 case fatality that are simply more or less prevalent in Appalachia, but not considered here. Proximity to Tennessee, which has had higher incidence and case fatality rates due to COVID-19, or to West Virginia, where incidence has been lower, might explain some regional differences observed in Kentucky.^14^ Another possibility is simply that the number of analytical units for the Appalachian region (n=18 county groups) is too few to provide reliable accuracy. It is notable, however, that a first draft of this article considered substantially fewer weeks of data on COVID-19 cases and deaths (analysis not shown), and the updated analysis shown here resulted in no major differences to interpretation.

In most cases, higher correlation coefficients were observed in both regions separately than when analyzed together, regardless of statistical significance. This is partially due to generally higher rates of the BRFSS conditions and behaviors in the Appalachian region, as observed in Table 1 and Figure 4. Note in all scatter plots that the Appalachian points are distributed among higher values on the x-axis, even though the fitted line can be similar, if less steep. This is why the fitted line appears flatter when both regions are analyzed together— the Appalachian values extend the overall point pattern farther into the lower-right quadrant of the graph, thereby obscuring the positive correlations in each region. This is most evident in the scatter plot for physical inactivity, where the correlation coefficients—and fitted line slopes—for each region, are similar, but Appalachia is shifted to the right due to generally higher rates of physical inactivity in that region. Future public health researchers should carefully consider the Appalachian region in epidemiologic analysis of COVID-19 outcomes relative to cardiovascular conditions or risk factors. Failure to stratify by—or adjust for—the Appalachian region in could bias toward null findings in population-level analyses such as this one. It is important to note, however, that the findings of this study also indicate the Appalachian region may have less relevance for this pandemic than other public health matters, such as cancer, diabetes, and heart disease.

This study is limited in some ways that merit careful consideration. First, using cumulative case and death counts to estimate case fatality is not optimal until an outbreak has concluded, which is certainly not the case for the COVID-19 pandemic as of this writing. A better estimate of case fatality would have used the sum of deaths and recovered individuals as the denominator but counts of recovered individuals were not available at the county level in the data set accessed for this study. It seems unlikely, however, that this issue would explain the notable differences that were observed between Appalachian and non-Appalachian regions.

The definition of the Appalachian region proposed by this work is also somewhat limiting in that it does not conform to the county-level designations from the ARC. Alternative analyses that included both additional and fewer county groups designated as Appalachian produced very similar results (analysis not shown), however, indicating that this study’s results are relatively robust to small changes in the Appalachian classification.

This analysis noted slightly lower but very similar COVID-19 case fatality in Appalachian Kentucky compared to the rest of the state, at least through February 28, 2021. Further, case fatality in the region was not as closely associated with important measures of population health as outside the region. The findings of this study are largely consistent with much of the recent research describing cardiovascular conditions and related behaviors as important risk factors for severe COVID-19 disease. There are, however, important differences between Appalachian and non-Appalachian regions which merit further research and should be considered when examining COVID-19 outcomes in Kentucky and several neighboring states.

**SUMMARY BOX**

**What is already known about this topic?** Chronic cardiovascular conditions are known to be associated with risk of severe COVID-19 symptoms or death.

**What is added by this report?** This report shows that COVID-19 case fatality rates are relatively low in Appalachian Kentucky, despite high prevalence of such conditions in the local population.

**What are the implications for future research?** The Appalachian region does not necessarily have high COVID-19 case fatality rates but may need to be considered separately in analysis of population health.

## Figures and Tables

**Figure 2a:**
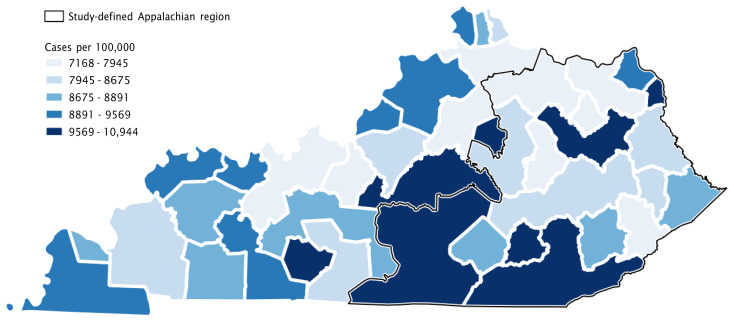
COVID-19 Incidence in Kentucky As of 1 March 2012

**Figure 2b:**
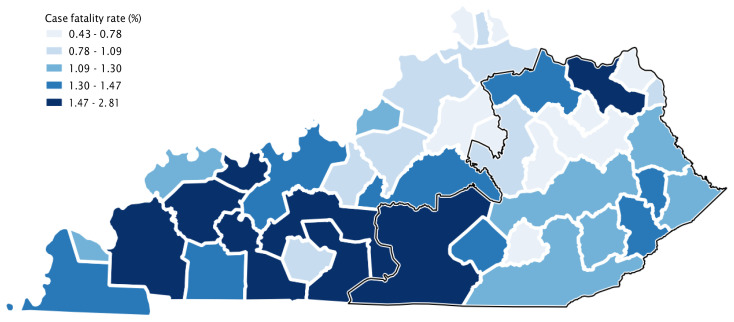
COVID-19 Case Fatality by County Group

**Table 1:** Chronic conditions and behaviors (BRFSS) and COVID-19 incidence and case fatality rates among Appalachian and non-Appalachian county groups

	Non-Appalachian (n=23)					Appalachian (n=18)					
	Mean	SD	Med	Min	Max	Mean	SD	Med	Min	Max	p-value
Respondents per county group	629	628	499	278	3423	541	216	501	248	1053	0.58
Current asthma	14.3	3.0	14.4	9.2	20.1	17.8	3.6	18.9	10.0	23.8	<0.01
Diabetes*	13.4	2.6	13.9	8.2	17.9	17.4	4.5	16.8	9.5	28.6	<0.01
Influenza vaccination	39.8	3.4	40.1	32.2	45.6	39.4	5.1	39.5	30.9	49.6	0.74
Hypertension	39.2	5.0	38.8	31.4	50.8	48.4	6.8	49.7	36.7	60.4	<0.01
Obesity	35.3	4.1	34.2	29.1	45.7	41.3	3.3	41.5	34.6	48.0	<0.01
Have a personal doctor	81.7	3.8	81.4	72.3	89.4	85.1	3.2	84.9	80.4	90.4	<0.01
Physical inactivity	33.1	5.0	32.4	24.6	40.2	38.9	4.4	38.1	29.1	45.9	<0.01
Cigarette smoking	24.4	4.8	25.8	15.8	33.6	27.0	4.3	27.5	17.5	34.8	0.08
COVID-19 incidence	8839	911	8879	7168	10,944	8797	928	8705	7552	10,493	0.89
COVID-19 case fatality*	1.26	0.53	1.20	0.61	2.81	1.14	0.40	1.18	0.43	1.97	0.58
